# Quantitative and Qualitative Aspects of Composite Action of Concrete and Dispersion-Reinforcing Fiber

**DOI:** 10.3390/polym14040682

**Published:** 2022-02-11

**Authors:** Sergey A. Stel’makh, Evgenii M. Shcherban’, Alexey Beskopylny, Levon R. Mailyan, Besarion Meskhi, Valery Varavka

**Affiliations:** 1 Department of Engineering Geology, Bases, and Foundations, Don State Technical University, 344003 Rostov-on-Don, Russia; sergej.stelmax@mail.ru (S.A.S.); au-geen@mail.ru (E.M.S.); 2 Department of Transport Systems, Faculty of Roads and Transport Systems, Don State Technical University, 344003 Rostov-on-Don, Russia; 3 Department of Roads, Don State Technical University, 344003 Rostov-on-Don, Russia; lrm@aaanet.ru; 4 Department of Life Safety and Environmental Protection, Faculty of Life Safety and Environmental Engineering, Don State Technical University, 344003 Rostov-on-Don, Russia; reception@donstu.ru; 5 Research and Education Center “Materials”, Don State Technical University, Gagarin sq. 1, 344003 Rostov-on-Don, Russia; varavkavn@gmail.com

**Keywords:** glass fiber, dispersion-reinforcing fiber, fiber concrete, concrete matrix, strength characteristics, strain characteristics

## Abstract

The interest in using polymer-dispersed reinforcement in the construction industry in the context of sustainability has led to significant research on this scientific problem. The article is devoted to studying the processes of fiber interaction depending on its dispersion and the concrete matrix, and their combined contact work during the formation of a concrete structure, work under stresses arising in a concrete body, and during a collapse. The physical and mechanical processes of deformation and destruction of the “matrix–fiber” system were studied using high-precision microscopic equipment, and the nature of the work and deformation of fibers in concrete were revealed. The work aimed to establish and characterize the quantitative and qualitative aspects of the concrete matrix and dispersion-reinforcing fiber combined work. It was established that the best values of the adhesion index were observed at a volume content of fiber in the amount of 2% by weight of cement, regardless of the type of dispersion-reinforcing fiber. It was shown that the microstructure of polydispersion-reinforced fiber-cement specimens was denser, and microcracks formed during fracture in polydispersion-reinforced specimens had a smaller opening width. It was established that polydispersion-reinforced concrete had higher values of strength (up to 126%) and deformation (up to 296%) characteristics compared to monodispersion fiber-reinforced concrete.

## 1. Introduction

Current trends in the development of architectural, planning, and design solutions for building and structure technologies for the construction of objects for various purposes require radical improvements in the physical, mechanical, and operational characteristics of products and structures, reducing material, labor, and energy recourses. In this regard, the theoretical substantiation and development of new effective materials based on polymers, their testing methods, calculation, and design of structures are relevant and of scientific interest. One of the most promising among modern building materials is fiber-reinforced concrete, which contributes to the solution of this problem. At the same time, the scope of the practical application of this composite material can be significantly increased in the case of obtaining more detailed and reliable information about its various and unique properties and characteristics. A severe disadvantage of concrete is fragility in fracture, which occurs as a result of the almost instantaneous propagation of the main crack after slight plastic deformation. However, in the case of ideally brittle fractures, it is possible to reconstruct a sample of the same sizes from parts obtained during testing without gaps between them. A brittle fracture most often occurs along certain crystallographic planes inside grains. This so-called intragranular fracture can be realized by cleavage and shearing, which differ in the type of fracture surfaces [1,2,3,4,5].

As studies [6,7,8,9] of composite materials on brittle matrices have shown, the effect of reinforcing fibers is most effectively manifested at the stage of formation and opening of cracks. In this case, one of the determining factors in the behavior of each fiber is its orientation relative to the crack front. The methods of dispersed reinforcement provide for the possibility of obtaining directed and arbitrary (free) orientation of fibers in the volume of concrete. Directional orientation is realized mainly when using continuous threads, ropes, various kinds of woven and non-woven nets, sparse fabrics, and other similar materials. A similar orientation can also be carried out when reinforcing concrete with short fibers, particularly steel fibers, when molding products, for example, in a magnetic field [4,9]. Arbitrary orientation is carried out, as a rule, with short fibers, but in this case, roll materials in the form of canvases, mats, and veils, in which the fibers do not have an organized weave, can also be used. In practice, various types of arbitrary orientation can be implemented in structures [10,11,12,13,14,15]. Plane-arbitrary orientation is characterized by an equiprobable and unlimited (free and chaotic) distribution of fibers in a plane (in two-dimensional space). Dispersed reinforcement, in this case, is realized mainly in thin-walled products in the form of flat sheets, plates, and elements with a curvilinear shape. The thickness of the products in this case, as a rule, is less than the length of the fibers used, while the angles of inclination of the fiber concerning the surface of the products are relatively small [1,3,16]. Volumetric arbitrary orientation is characterized by an equiprobable and unlimited (free and chaotic) distribution of short reinforcing fibers throughout the entire volume of concrete (in three-dimensional space). The angles of inclination of the fibers in relation to the surface of the products are from 0 to 90°, and the dimensions of the products in all directions significantly exceed the length of the fibers. Constrained-arbitrary orientation occurs when at least two geometric parameters of structural elements, for example, their height and width, are limited in size, which hinders the freedom of arbitrary orientation of the reinforcing fibers in the bulk of concrete. A similar situation is observed with dispersed reinforcement of beams, plate edges, various kinds of bridges, etc. The smaller the cross-sectional dimensions of the products, the more limited the possibilities of the free orientation of the reinforcing fibers. The analysis shows that the effect of constraining the orientation of the fibers is manifested mainly in those cases when the corresponding dimensions of the products exceed the length of the reinforcing fibers by no more than five times. With larger cross-sectional dimensions of products, the effect of restraint is noticeably reduced, and the parameters of fiber orientation in a concrete matrix, in this case, approach the parameters of plane-arbitrary or volume-arbitrary reinforcement [1,3,14,15,16,17].

The degree of all these changes in material properties and the structures’ characteristics is mainly determined by the type and properties of the fibers and concrete used and the degree of interaction between them. Thus, a close relationship of reinforcing fibers with a concrete matrix, in addition to its activity, is provided by the length of the boundaries, which depends on the degree of saturation of concrete with fibers, their geometric characteristics, and surface energy [7,10,11,12,13]. Considering this, it is logical to assume that the greatest positive effects in improving the structure and properties of fiber-reinforced concrete can be achieved as a result of polydispersed reinforcement with an optimal combination of two or more types of fibers with different geometric dimensions. The purpose of polydispersed reinforcement is to create a material that, while maintaining and increasing the achieved advantages, would eliminate the disadvantages of the composite that occur in the variant of monodispersed reinforcement. Thus, the advantages of polydispersed reinforcement are as follows:

- Obtaining a composite with greater strength than with monodispersed reinforcement;
- Purposeful regulation of the complex of physical and mechanical properties of the composite over a broader range than with monodispersed reinforcement;
- The possibility of improving the indicators of durability and operational characteristics of the composite [14,15,16,17,18,19,20,21]. A number of works [18,19,20] considered the mechanical properties of concrete, dispersion-reinforced with various types of fibers. According to the results of studies carried out in [18], it was found that concrete reinforcement with a combination of aramid and carbon fibers has a significant effect on the mechanical strength of reinforced concrete. In general, the mechanical strength of concrete reinforced with a combination of aramid and carbon fibers was better than that of concrete with only Kevlar or carbon fibers. In [19], the combined effect of nylon and jute fibers on density, water absorption, compression, tension, flexural strength, and concrete shrinkage upon drying was investigated. The results showed that concrete with 1% nylon and jute fibers together by volume fraction showed the maximum increase in compressive strength, tensile strength, and flexural strength compared to the control concrete mixture. However, the water absorption of concrete increased with the increasing content of nylon and jute fiber, and concrete drying shrinkage was reduced by adding nylon and jute fibers. In [20], the authors investigated the effect of the combined introduction of steel and polypropylene fibers and their content on the rheological and mechanical properties of self-compacting concrete. The results showed that the workability of self-compacting concrete decreases due to an increase in the content of steel or polypropylene fibers; however, the rheological characteristics of the laid mixtures met the recommendations of the European Directives for self-compacting concrete for fresh concrete, and the tensile, flexural, and shear strength during splitting was increased due to increasing the fiber content [20]. The reviews in [21,22,23,24,25] showed that the introduction of fibers increases the crack resistance of concrete, that is, its ability to resist the development of cracks. For example, in [21], the mechanism of fracture of cement composites was studied, and the effects of fiber on the fracture properties of a cement composite were determined. It was found that the fracture properties of the cement composite can be significantly improved by adding 1.5–2% PVA fiber or 4% steel fiber [21].

Dispersion-reinforcing fibers have also found wide application in cellular concrete technology [26,27,28,29]. For example, in [26], the authors studied the effect of dispersed reinforcement in the production of non-autoclaved cellular foam concrete. Dispersed reinforcement has a positive effect on increasing the bearing capacity of a freshly prepared foam concrete mixture, which leads to the elimination of destructive processes and an increase in dimensional stability. In [27], studies were carried out on lightweight cellular concrete for structural use in brickwork. The authors found that the addition of synthetic fibers improves the strength characteristics of cellular concrete. In [28], the authors studied the behavior of cellular concrete reinforced with hybrid-synthetic fiber under uniaxial tension. The inclusion of reinforcing fibers prevents premature failure and improves stiffness and ductility after cracking, and limited crack localization and improved ductility are also recorded due to the addition of fibers. In [29], a model is presented for studying the permeability and Kozeny–Carman (KC) constants of fibrous porous media consisting of solid particles and porous fibers. An increase in fiber diameter leads to an increase in the absolute permeability of fibrous porous media. The presented fractal model establishes the relationship between the KC constant and permeability with the microstructural parameters of a fibrous porous medium and makes it possible to facilitate understanding of the mechanism of fluid transfer through a fibrous porous medium [29].

The conducted literature review, firstly, showed that the actual direction of building science is the study of fiber reinforcement of concrete. At the same time, all the work carried out in this direction is quite relevant due to the universality of the concept of “fiber-reinforced concrete” and the expanded number of technological and recipe possibilities for controlling the properties of various concretes due to numerous combinations and techniques associated with dispersed reinforcement. Furthermore, it should be noted that many studies are closely related to each other due to the fact that the influence of various parameters of dispersed reinforcement should often be considered in a synergistic sense. For example, this can be a simultaneous study of the influence on the properties of fiber-reinforced concrete of both dispersion parameters, that is, the geometry and quantitative aspect of the fibers, and its structural aspect, that is, the percentage of reinforcement, and, finally, from the point of view of the qualitative composition, that is, the material from which the dispersion-reinforcing fibers are made. In this regard, certain scientific deficits require additional research in terms of quantitative and qualitative aspects, and this requires high-precision, microscopic, and other analytical equipment, as well as certain methodological tools to establish specific, measurable characteristics of work, both qualitative and quantitative, in the joint interaction of the matrix of cement stone and concrete as a whole with dispersion-reinforcing fibers filling the concrete structure. In addition, the literature review showed that data on the effect of polydispersed reinforcement on the strength of fiber-reinforced concrete is much less than that of monodispersed reinforcement. However, these data are sufficient to conclude that it is possible to ensure regulation of material properties within broader limits than with monodispersed reinforcement [30,31,32,33].

From the point of view of fundamental science, the question of the joint work of concrete and fiber dispersion-reinforcing fiber remains completely unexplored at present. The processes occurring at the interface between the cement stone, aggregate, if any, and fiber have not been studied; the issue of fiber deformation is of interest. If the destruction and rupture of fibers have been studied to a more or less certain extent, then the deformations of fiber, which are at the micro-level a reinforcing rod with a high degree of flexibility, have not been studied in detail. Therefore, it is essential to obtain fundamental knowledge about the processes of interaction of fiber and a concrete matrix, their joint work in terms of contact, during the formation of a concrete structure, during operation, under stresses arising in the concrete body, and destruction, which is most interesting and is an unexplored physical–mechanical process, which can be investigated only by using high-precision microscopic equipment. The motivation for the study was the reasonable relevance of a new generation of fiber-reinforced concrete due to the increased requirements for construction objects and building materials. The study aimed to determine the qualitative and quantitative aspects of concrete and fiber combined work with monodispersed and polydispersed reinforcement. At the same time, the achievement of the goal was methodologically planned as the implementation of a new approach to research based on the formula, technological and microstructural aspects, and consideration of the synergistic system of the composite material “cement matrix–concrete matrix–fiber”. Thus, the tasks of the study include microscopic analysis of the contact zone and the nature of deformation and destruction of fiber and a concrete matrix during the destruction of fiber-reinforced concrete composites, identifying the possibility of a qualitative and quantitative assessment of such interaction, and then conducting a quantitative assessment of this work and a qualitative analysis with the characterization and construction of a picture of the physical and mechanical processes occurring during the operation of fiber-reinforced concrete elements.

## 2. Materials and Methods

To begin to determine the order of the program and the plan of experimental and analytical studies, one should determine the methodological apparatus of the study and consider in-depth the main aspects that are touched upon in this study.

Thus, from the point of view of methodology, as it was already established above in the literature review and analysis of the current state of the problem, there is a shortage of complex synergistic methods that simultaneously consider several aspects of the work of concrete and fiber. Thus, the methodological aspect consists, firstly, in considering fiber-reinforced concrete as a composite system consisting of a matrix of cement stone, which is part of another composite material—concrete, where it also acts as a matrix where the fillers in concrete are aggregates and fibers. In this case, the fiber is considered in direct interaction with the cement stone, with a matrix consisting exclusively of hardened, prohydrated cement. This approach is due to the possibility of additional microscopic examinations from the point of view of studying the behavior of fiber dispersion reinforcing fibers during operation under load and considering fibers as dispersed reinforcement at the micro-level. The research methodology from the point of view of the formulation is to select, based on the available literature data, the initial components of fiber-reinforced concrete in order to direct the study in a specific measurable direction, namely, the study of the behavior of fibers under load in concrete. The most rational raw material components (binder, filler, aggregator) were those components that are listed below.

From the point of view of technology, the sequence of introducing the components was determined, the order of their mixing was determined, and the subsequent homogenization was carried out in accordance with the previously-developed most effective proposals of the authors indicated in the “Introduction”.

From the point of view of the study, a methodology was developed that consists of the manufacture and proper curing of prototypes of cement stone reinforced with dispersion fiber, intended for microstructural studies and their analysis, and coarse concrete, intended for understanding the quantitative characteristics of work—improved concrete in general.

During the research, we used Portland cement, grade PC 500 D0 (Oskolcement OJSC, Stary Oskol, Russia) [34], without additives, the physical and mechanical characteristics and chemical composition of which are presented in Table 1.

Granite crushed stone (Pavlovsk nerud, Pavlovsk, Russia) was used as a large dense aggregate, the physical and mechanical characteristics of which are presented in Table 2. Quartz sand (Yuzhny GOK, Rostov-on-Don, Russia) was used as a dense fine aggregate, the physical characteristics of which are presented in Table 2.

Glass fiber (Armplast, Nizhniy Novgorod, Russia) [35] pretreated with surfactants was used as the dispersed reinforcement. Table 3 shows the physical and mechanical characteristics of the fiber used.

Fiber types A and B differed in geometric parameters and both were treated with surfactants.

To develop combinations of polydispersed reinforcement and predict their effectiveness, it seems appropriate to determine the amount of adhesion of glass fiber to the matrix.

Most often, the destruction of fiber-reinforced concrete occurs due to the pulling of fibers from the concrete as a result of a breakdown of the bond at the fiber–matrix interface. Thus, by increasing the adhesion strength of the fiber to the matrix, it is possible to maximize the use of strength properties of fiber, up to its rupture at the moment of destruction of the composite. The adhesion of the fiber to the concrete matrix of the composite is the result of the combined action of adhesion, friction, and mechanical engagement in the zone of their contact with the cement stone. The influence of each of these factors on the anchoring of fibers in the matrix can be different and depends on the composition, structure, and properties of the cement stone and the fibers’ material, shape, and size. Thus, the main mechanism for increasing the strength of dispersion-reinforced concrete is an increase in the adhesion strength of the reinforcing fiber with the concrete matrix of the composite [1].

Determination of the adhesion index of the fiber to the matrix consists of finding experimentally such a minimum percentage of concrete reinforcement, up to which the introduced fibers practically do not show a reinforcing effect, but after which a steady increase in the strength of the composite is observed [1]. Table 4 shows for comparison the minimum and maximum contents of fibers previously used by other researchers.

As a starting mixture, a cement paste of normal density is used. Then, reinforced and unreinforced samples of a certain composition are prepared from one batch, and according to the test results, a graph “Strength–volumetric content of fiber” is plotted. As for the adhesion characteristics of fibers with a cement stone from a dough of normal density [1], they are determined by the formula

(1) $$\tau =\frac{R_{fc}-3.5\cdot R_{ca}\cdot \mu_{\min}-\left(1-4.5\cdot \mu_{\min}\right)\cdot R_{cs}}{2\cdot \frac{l}{d}\mu_{\min}}$$

where $R_{fc}$, $R_{cs}$, and $R_{ca}=1.4\;R_{cs}$ are the tensile strength of fiber cement in bending, cement stone from dough of normal density, and contact zone; $\mu_{\min}$ is a coefficient of reinforcement (volumetric content of fibers); and *l* and *d* are the length and diameter, respectively, of the fiber [1].

The experimental research program is presented in Table 5.

One series of samples includes three beams with dimensions of 40 × 40 × 160 mm. Each series of specimens was tested for compression and flexural tension.

Based on the results of preliminary experimental studies, the most effective type of dispersed reinforcement and the reinforcement coefficient were determined.

The main experimental part included the manufacture of concrete and fiber-reinforced concrete samples to determine the following characteristics: cube compressive strength (cubes 100 × 100 × 100 mm, 3 pcs.); prismatic compressive strength and ultimate deformations during axial compression (prisms with dimensions 100 × 100 × 400 mm, 3 pcs.); tensile strength in bending (prisms with dimensions 100 × 100 × 400 mm, 3 pcs.); and axial tensile strength and ultimate deformations during axial tension (prisms with dimensions 100 × 100 × 400 mm, 3 pcs.).

The preparation of the fiber-reinforced concrete mixture was carried out in a BL–10 laboratory forced-action concrete mixer with the following sequence of loading the components: fine aggregate, Portland cement, and water. Upon reaching the homogenization of the mixture of the binder, grout, and fine aggregate, fiber was introduced into it. Mixing of the fiber cement–sand mixture was carried out for 3 min until the fiber was thoroughly distributed in the volume of the mixture, after which coarse aggregate was introduced into it. To compact the mixtures in the process of forming the samples, a standard vibrating platform was used, and the vibration time was 90 s. On the next day after molding, the samples were stripped and placed in a normal hardening chamber for 28 days until the design strength was achieved.

Compressive and tensile flexural strength tests were carried out in accordance with GOST 10180-2012 “Concretes. Methods for strength determination using reference specimens” [36] (ASTM C39/C39M-21).

The determination of the modulus of elasticity and prismatic strength was carried out in accordance with the requirements of GOST 24452-80 “Concretes. Methods of prismatic compressive strength, modulus of elasticity and Poisson’s ratio determination” [37] (ASTM C469/469M-14e1).

The measurements of the concrete deformations of the test prisms were carried out with a chain of strain gauges with a base of 50 mm and dial indicators with a graduation of 0.001 mm.

Experimental prisms were tested for axial compression and axial tension at a constant rate of deformation to obtain not only the strength and deformation characteristics of concrete, but also its full deformation diagrams σ–ε with descending branches.

The following were also used in this study: a BL-10 laboratory concrete mixer (LLC “ZZBO”, Zlatoust, Russia); an SMZh-539-220A laboratory vibrating platform (LLC “IMASH”, Armavir, Russia); hydraulic press IP–1000 (LLC NPK TEKHMASH, Neftekamsk, Russia); an R-50 tensile testing machine (LLC “IMASH”, Armavir, Russia); a 500 mm metal measuring ruler, laboratory scales, an NPL-1 device for measuring deviations from the plane, and an NPR-1 device for measuring deviations from perpendicularity (NPO LABORKOMPLEKT, Moscow, Russia) [38,39,40,41].

The study of the microstructure of polydispersion-reinforced and monodispersion-reinforced fiber cement samples was carried out by scanning electron microscopy (SEM).

The study of the microstructure of a dispersion-reinforced cement stone to determine the size of microcracks, the shape of structural elements, and their orientation in space was carried out using a ZEISS CrossBeam 340 double-beam scanning electron/ion microscope equipped with an Oxford Instruments X-Max 80 X-ray microanalyzer (Carl Zeiss Microscopy GmbH (Factory), Jena, Germany).

## 3. Results

### 3.1. Investigation of the Strength Characteristics of Monodispersion-Reinforced and Polydispersion-Reinforced Fiber Cement Specimens

**Figure 1:**
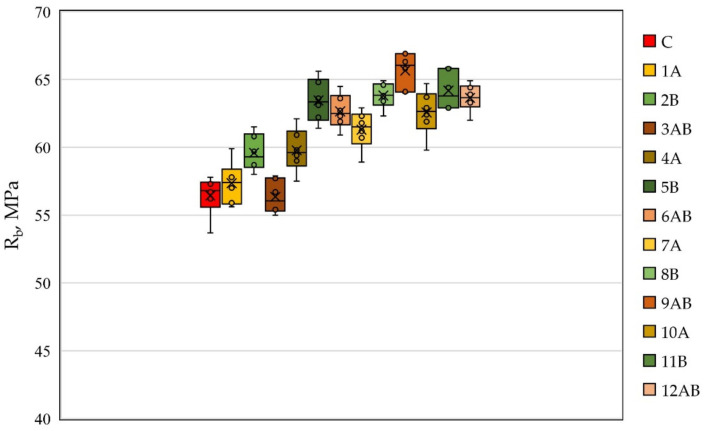
The compressive strength of fiber cement specimens depending on the type of dispersed reinforcement.

**Figure 2:**
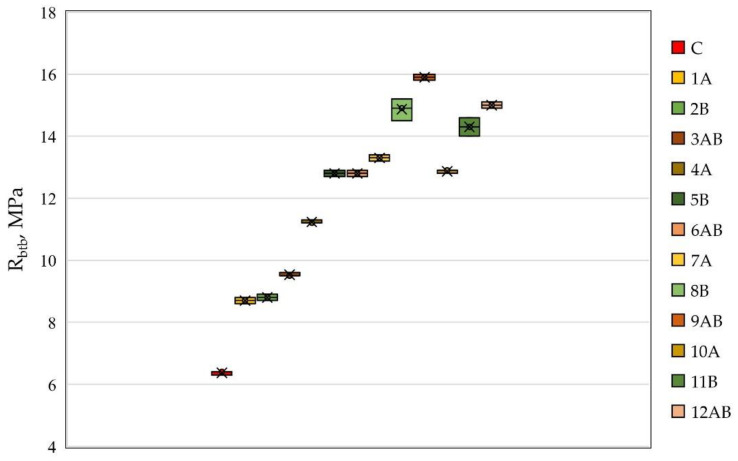
The tensile strength of fiber cement specimens depending on the type of dispersed reinforcement.

The results obtained from preliminary tests of prototypes of fiber cement beams are presented in Figure 1 and Figure 2.

Figure 1 shows the values of compressive strength for all test compositions of fiber cement samples. As can be seen, the lowest value of the compressive strength of 56.4 MPa was observed for specimens of composition type C. Compressive strengths for compositions of type 1A, 4A, 7A, and 10A were, respectively, 57.4 MPa, 59.8 MPa, 61.3 MPa, and 62.6 MPa. The values of compressive strength for compositions of type 2B, 5B, 8B, and 11B were, respectively, 59.6 MPa, 63.5 MPa, 63.8 MPa, and 64.2 MPa.

As for the polydispersion-reinforced fiber-cement specimens, their compressive strength values were higher compared to the strength of monodispersion-reinforced specimens. For example, the compressive strength values for compositions of the 3AB, 6AB, 9AB, and 12AB types were 56.4 MPa, 62.7 MPa, 65.7 MPa, and 63.7 MPa, respectively.

It should also be noted that the effects of the volumetric content of fiber in the composition of the cement matrix, both in the case of monodispersed reinforcement with type A fibers and monodispersed reinforcement with type B fibers, were similar, namely, with an increase in the percentage of reinforcement from 1 to 4%, an increase in strength was observed. In the case of polydispersed reinforcement, with the introduction of type A and B fibers with ratios of 0.5 and 0.5% and 1 and 1%, an increase in strength characteristics was observed; with the introduction of these fibers in a ratio of 1.5% and 1.5%, the maximum strength value was recorded; and with the introduction of these fibers in the amount of 2 and 2%, a decrease in strength was observed.

From Figure 2, it follows that the minimum value of the tensile strength in bending was observed in the same way as for the compressive strength for composition C and was 6.4 MPa. The maximum tensile strength was recorded for polydispersion-reinforced fiber cement specimens with a volumetric content of type A and B fibers with a ratio of 1.5 and 1.5% and was equal to 15.9 MPa. For fiber cement compositions of type 1A, 4A, 7A, and 10A, the tensile strength values in bending were 8.7 MPa, 11.2 MPa, 13.3 MPa, and 12.9 MPa, respectively. For fiber cement compositions of type 2B, 5B, 8B, and 11B, the values of tensile strength in bending were 8.8 MPa, 12.8 MPa, 14.9 MPa, and 14.3 MPa, respectively. As can be seen, in the case of monodispersed reinforcement with type B fibers, the tensile strength in bending was higher in comparison with the samples reinforced with type A fibers. However, in both cases, the tendency for the strength to change depending on the volumetric content of fibers was the same. Thus, with the introduction of fibers in the amount of 1, 2, and 3%, the strength increased, and with the introduction of 4%, it decreased. This tendency to change the tensile strength in bending was equivalent to that that of polydispersed reinforcement. The tensile bending strength values for compositions of the 3AB, 6AB, 9AB, and 12AB types were 9.5 MPa, 12.8 MPa, 15.9 MPa, and 15 MPa, respectively.

**Figure 3:**
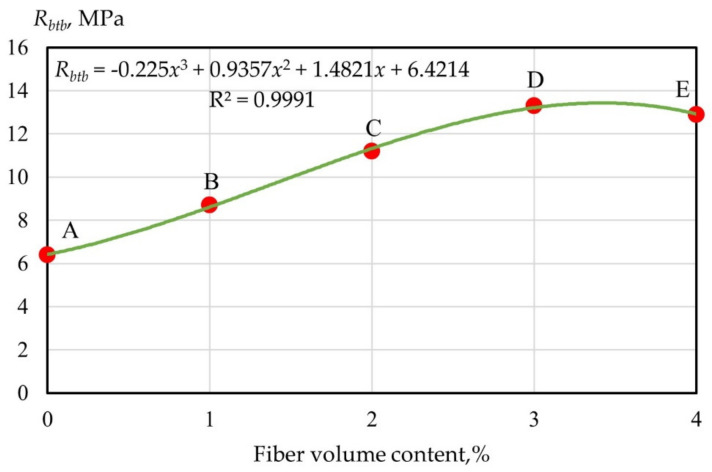
Dependence of the strength of monodispersion-reinforced fiber cement specimens on the volumetric content of glass fiber type A.

**Figure 4:**
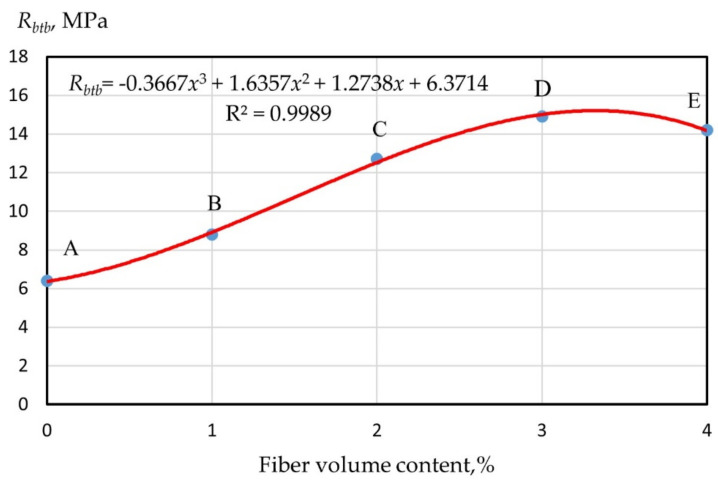
Dependence of the strength of monodispersion-reinforced fiber cement specimens on the volumetric content of glass fiber type B.

Let us consider the nature of the change in the tensile strength in bending of fiber cement specimens reinforced with glass fiber in the range from 0 to 4% (Figure 3 and Figure 4). Dependence of the strength of monodispersion-reinforced fiber cement specimens on the volumetric content of glass fiber type A and B and polydispersion FRC are presented in (2)–(4). The values of the determination coefficients given in Equations (2)–(4) showed a good relationship between the regression curve and the data points of the strength characteristics of geopolymer mixtures.

(2) $$R_{btb}^{AB}=-0.35x^{3}+1.5143x^{2}+1.6929x+6.4486;\;R^{2}=0.9974$$

(3) $$R_{btb}^{B}=-0.3667x^{3}+1.6357x^{2}+1.2738x+6.3714;\;R^{2}=0.9989$$

(4) $$R_{btb}^{A}=-0.225x^{3}+0.9357x^{2}+1.4821x+6.4214;\;R^{2}=0.9991$$

From Figure 3 and Figure 4, it follows that within the AB section, the strength of dispersion-reinforced samples did not differ significantly from the strength of the cement matrix, which was determined by the low saturation of concrete with fibers when they were far enough away from each other and practically did not interact. This area can be figuratively called the “zone of diffuse reinforcement”.

Point B corresponds to the situation when from the moment cracks appeared in the cement matrix, the applied load was absorbed by the fiber and provided the bearing capacity.

The BC section defines the so-called “concentrated reinforcement zone”, and point C is the moment of merging of the contact zones that arose in the process of structure formation near the “fiber–matrix” interface, and thus the formation of a bulk fiber cement framework.

Section CD characterizes a further, and more intensive, increase in strength in the “zone of carcass reinforcement”, which is the result of the consolidation of the cement stone between the fibers. Point D corresponds to the achievement of the maximum strength, after which its decrease was observed, caused by a decrease in the thickness of the matrix layer so that the material tended to delaminate.

**Figure 5:**
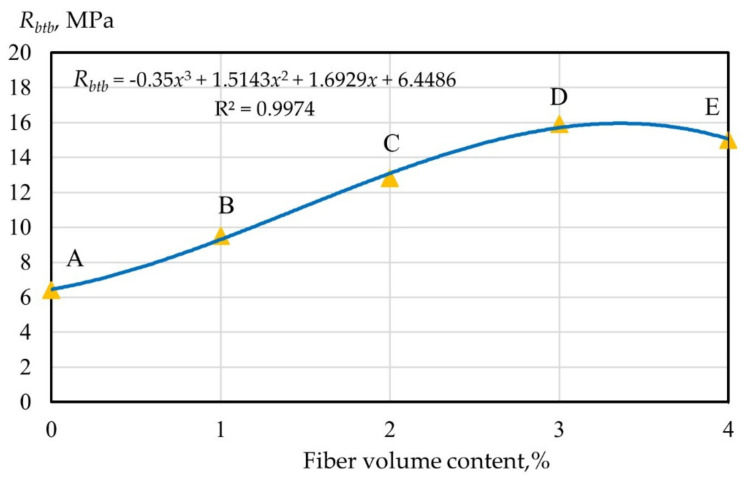
Dependence of the strength of polydispersion fiber-reinforced concrete depending on the volumetric content of glass fiber.

The strength of polydispersion-reinforced specimens changed in a similar way (Figure 5).

An analysis of literary sources made it possible to establish that most often, the volume content of glass fibers in fiber-reinforced concrete is 1.5–3.0% [6,7,8,11,13,30,31,32,33]. The dependence of the strength of fiber-reinforced concrete on the volume percentage of reinforcement is linear. However, it is known that with an increase in the degree of saturation of the concrete mixture with reinforcing fiber above a certain critical limit, the nature of the dependence changes. Thus, in works [1,2], a nonlinear change in the strength of the composite was experimentally established and theoretically substantiated depending on the limiting volumetric saturation of concrete with reinforcing fiber. Three characteristic values of the limits of bulk reinforcement were identified [1,2]. A comprehensive study comparing several types of fiber was also carried out, and their various combinations with polydispersed reinforcement determined the most rational concentrations of each type of fiber with monodispersed reinforcement and their optimal concentrations with polydispersed reinforcement.

Thus, according to the results of preliminary experimental studies, the indicator of adhesion of glass fiber to a cement stone for monodispersion-reinforced samples was calculated (Table 6).

Analyzing the calculated values of the adhesion index of the fiber to the cement stone, it can be seen that the values of the adhesion to the cement matrix for the compositions of type A were higher than those for the compositions of type B. In both cases, the best values of the adhesion index were observed with a fiber volume content of 2% by weight of cement.

### 3.2. Investigation of the Microstructure of Polydispersion-Reinforced and Monodispersion-Reinforced Fiber Cement Samples

**Figure 6:**
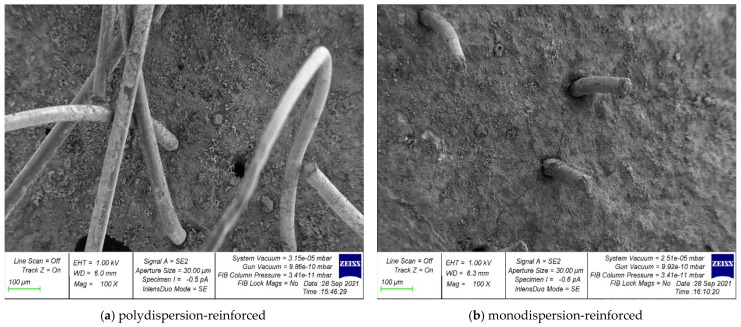
Microstructure of dispersion-reinforced fiber cement samples.

**Figure 7:**
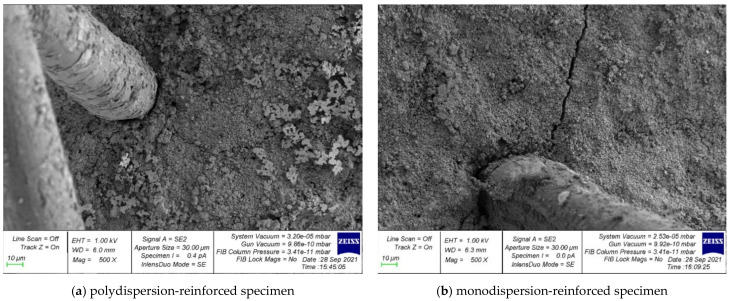
Crack formation in the “fiber-contact zone–cement matrix” system.

**Figure 8:**
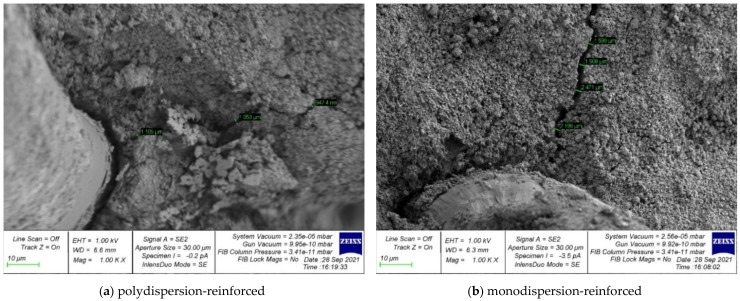
The microstructure of cracks in dispersion-reinforced fiber cement specimens.

**Figure 9:**
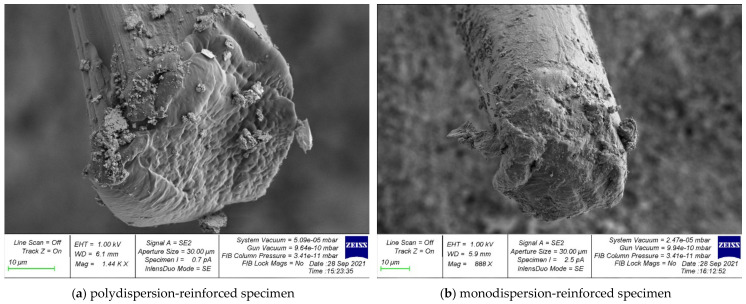
Surface analysis of broken fiber.

**Figure 10:**
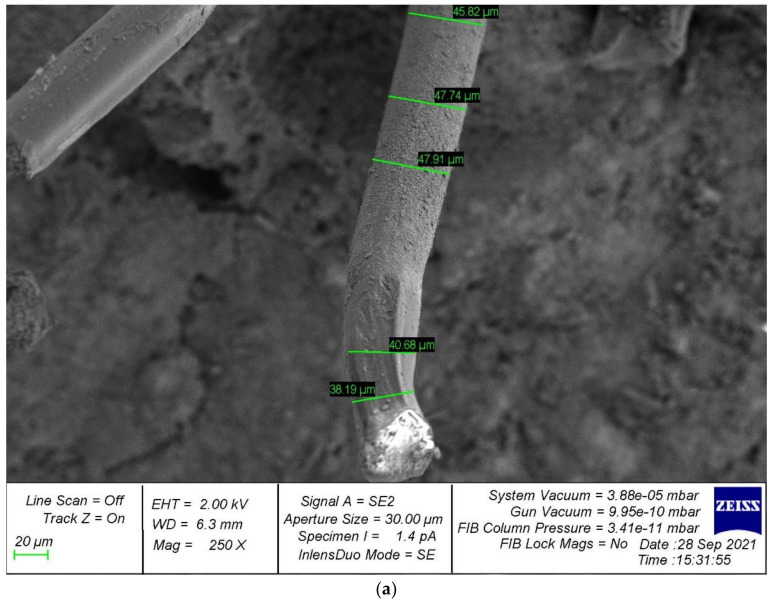

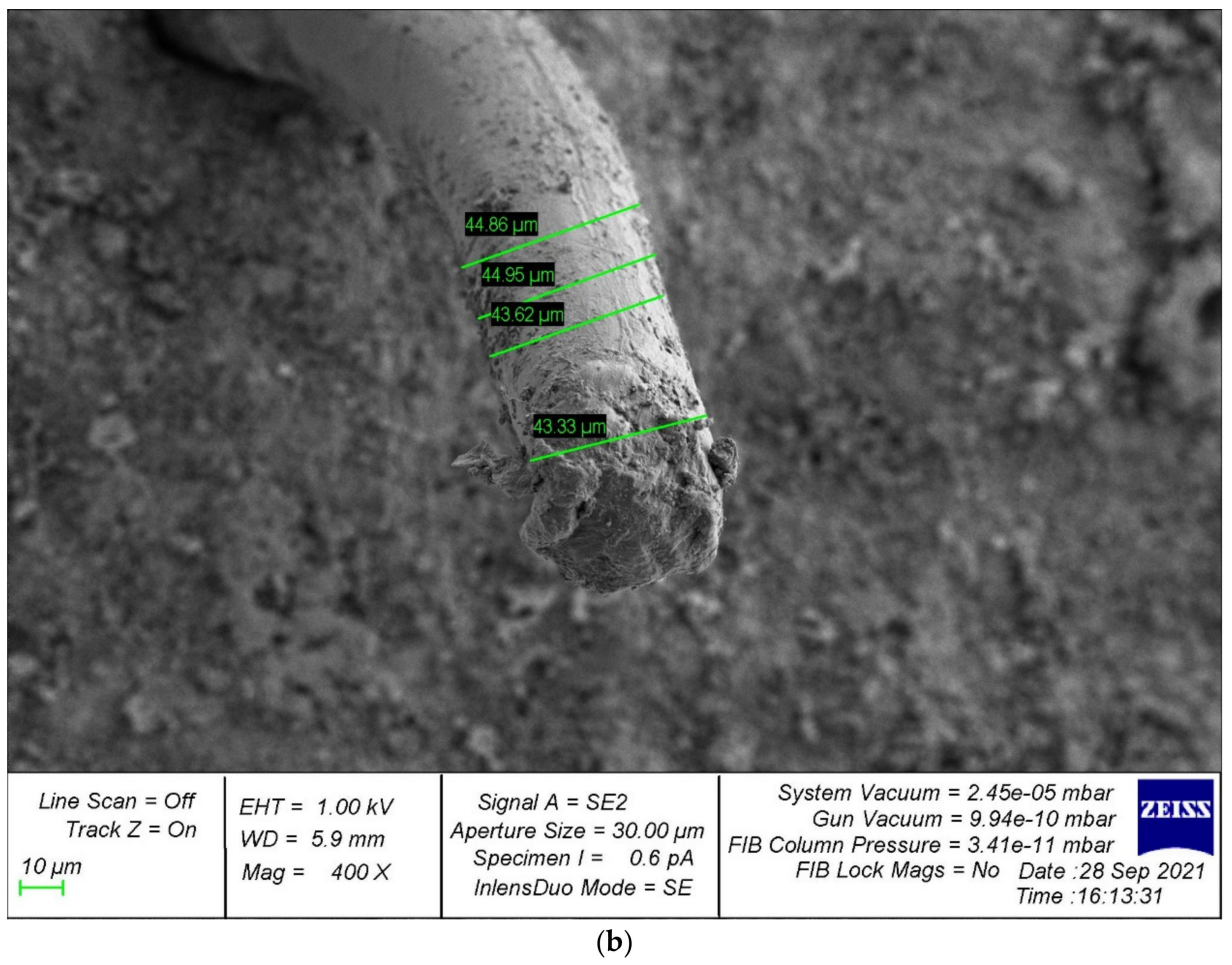
Analysis of the deformation of the fiber after fracture: (**a**) polydispersion-reinforced specimen; (**b**) monodispersion-reinforced specimen.

Photographs of the microstructure of polydispersion-reinforced and monodispersion-reinforced fiber cement samples are shown in Figure 6, Figure 7, Figure 8, Figure 9 and Figure 10.

Analyzing the photographs of the microstructures of the dispersion-reinforced fiber-cement samples presented in Figure 6, Figure 7 and Figure 8, we see that on the surface of the cement matrix of the polydispersion-reinforced samples, there was a smaller number of microcracks formed during the destruction of the test samples. In addition, for polydispersion-reinforced samples, a smaller width of the opening of microcracks was characteristic. Figure 8 shows that the average width of their opening for polydispersion-reinforced specimens was significantly lower than for monodispersion-reinforced specimens.

As an analysis of the photographs of the microstructure in Figure 9 and Figure 10, we will focus on the metamorphoses that occur with the fibrous fiber. There are two types of reinforcements: polydispersion (Figure 9a and Figure 10a), and monodispersion (Figure 9b and Figure 10b). Paying attention to the changes in the properties of the fibers after destruction, it should be noted that the fiber turned out to be the most unstable in the body of the cement matrix with monodispersed reinforcement. That is, we observed fiber pull-out, a large number of cracks, and intense cracking, which ultimately did not allow the fiber to be retained in the body of the cement matrix and reduced the ability to anchor. This problem was solved by polydispersed reinforcement, in which we saw fewer cracks, and in the case of the fiber itself, traces of its deformation. That is, it was the fiber that resisted and reinforces the cement matrix as a reinforcement sample and was not pulled out of the cement body and, thereby, strengthened the strong contact zone between the cement matrix and the reinforcing fiber.

Thus, it can be concluded that with polydispersed reinforcement, we first of all strengthened the “cement matrix–fiber” contact zone, and with monodispersed reinforcement, this zone was not reinforced, pulling out occurred at lower loads, the fiber was less resistant to pulling out, and thus the overall effect of the use of fiber dispersion-reinforcing fiber was diminished.

### 3.3. Influence of the Type of Dispersed Reinforcement on the Strength and Deformation Characteristics of Fiber-Reinforced Concrete

Heavy concrete of class B30 with the required workability grade P1 (cone draft of 1–4 cm) was designed as a control composition for the manufacture of test concrete samples. The content of coarse aggregate fractions is represented by the following ratio: 60%—fraction 10–20 mm; 40%—fraction 5–10 mm. The parameters of the composition of the concrete mixture obtained as a result of calculations are shown in Table 7.

Polydispersion-reinforced fiber-reinforced concrete samples were made from a similar composition of a concrete mixture with the addition of a combination of glass dispersion-reinforcing fibers in the amount of 3% of the cement mass, where one half was represented by fibers with a length of 6 mm and a diameter of 9 microns, and the other half was represented by fibers with a length of 12 mm and a diameter of 13 microns. The test results of concrete and fiber-reinforced concrete (FRC) samples of the main experimental part are presented in Table 8.

After analyzing the data presented in Table 8, it was found that the maximum values of strength and deformation characteristics were recorded for polydispersion-reinforced fiber-reinforced concrete.

Thus, the increase in cube compressive strength in polydispersion-reinforced fiber-reinforced concrete specimens in comparison with specimens from heavy concrete was 10%, the increase in prismatic compressive strength was 11%, tensile strength in bending increased by 126%, axial tensile strength increased by 45%, ultimate deformations under axial compression increased by 37%, ultimate deformations under axial tension increased by 296%, and the value of the elastic modulus increased by 12%.

Analysis of cracking and destruction showed a significant difference in the behavior of unreinforced and dispersion-reinforced concrete (Figure S1).

Unreinforced concrete collapsed with the formation of the first signs of cracking on the lateral faces of the specimen shortly before destruction, which occurred with the formation of a main vertical crack with minimal branching.

Polydispersion-reinforced concrete collapsed with the formation of multiple cracks with their characteristic branching, which indicates its high viscosity, as well as a higher energy consumption of the destruction process, which, in general, predetermines the high impact endurance of fiber-reinforced concrete in relation to the original unreinforced concrete.

**Figure 11:**
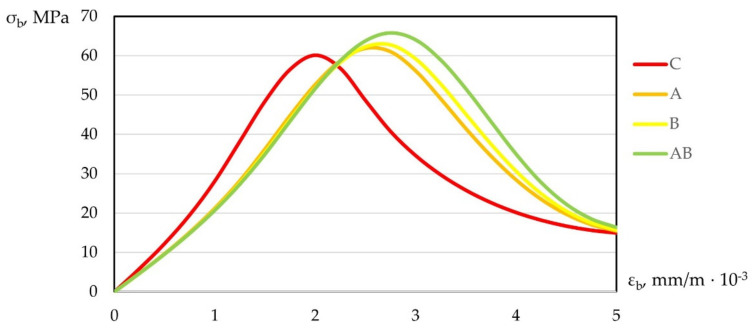
Diagram of “stress–strain” under compression.

**Figure 12:**
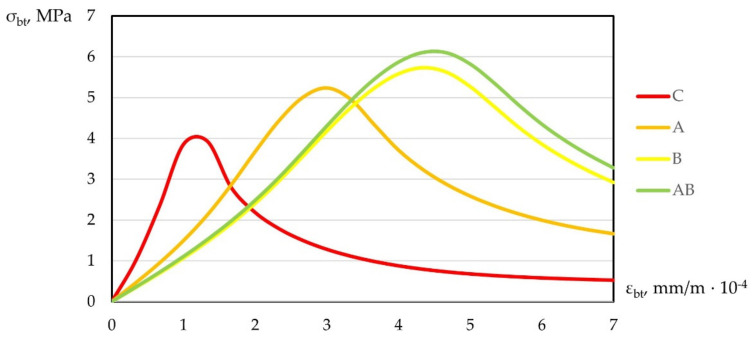
Diagram of “stress–strain” under tension.

In addition, according to the test results, compression diagrams $\varepsilon_{b}-\sigma_{b}$ and tension $\varepsilon_{bt}-\sigma_{bt}$ were plotted. Graphical dependences of “stress–strain” are shown in Figure 11 and Figure 12.

As can be seen from Figure 11 and Figure 12, under compression and tension, polydispersion-reinforced fiber concrete had the highest peak, shifted up and to the right relative to the peak of the heavy concrete diagram. A similar picture was observed with monodispersion-reinforced concrete.

Analyzing changes in deformation diagrams for various types of studied compositions, one can note the practical applicability of the data obtained. Taking into account the change in deformability and giving the material a more viscous character of destruction, creating a damping effect, we had the opportunity to control the properties by changing the qualitative and quantitative structures of concrete. Using fiber reinforcement, we achieved greater operational versatility of the produced concrete and expanded practical applicability in flexible construction requiring new approaches.

In general, the fracture toughness of the composite depends on the type and properties of the initial components and the volume ratio between them, mainly on the degree of saturation of the matrix with reinforcing fibers, and is ultimately determined by the strength of the phase boundaries. In this case, if the length of the fibers is greater than a certain critical length (*l_f_ > l_cr.f_*), then most of them will break at the moment of failure of the composite, and the fracture toughness will be low, and if (*l_f_ < l_cr.f_*), then the main contribution to the resistance of the composite to destruction will be made by the energy expended on pulling the fibers from the matrix.

## 4. Discussion

With polydispersed reinforcement, fibers of various geometrical parameters provide the formation of spatial cells at different levels of the concrete structure. Larger cells are superimposed on smaller ones, while the sizes of structural cells at each level depend on the reinforcement parameters. Thus, smaller fibers will be in the cramped conditions of larger cells, and larger fibers are placed in a composite matrix with micro reinforcement. In this case, microfibers prevent the development of micro defects at the cracking stage, being in the contact zone of fibers of a larger diameter, and reducing the stress concentration, thereby facilitating their redistribution to a larger volume of concrete. In this variant, the adhesion strength of the fibers of a larger diameter with the matrix increases, which ultimately makes it possible to achieve an increase in the strength of the composite [6,7,8].

However, this explanation of the structure formation of polydispersion-reinforced fiber-reinforced concrete gives a very idealized notion of its structure, which is based on the classical laws of composite materials, but at the same time does not take into account the important structure-forming role and originality of the concrete matrix, and also practically does not affect the role of the contact zone at the phase boundary “fiber-matrix“, the state of which largely determines the most important characteristics of dispersion-reinforced concrete [31].

In order to fully assess the scientific novelty and practical significance of the study, a comparative analysis with the results of other authors should be performed.

Let us conditionally divide the conducted research into two directions: fundamental science, and applied research. From the point of view of fundamental science, aspects of the operation of fiber in a concrete matrix were previously studied. Thus, in several works, various types of fiber [6,7,8,11,19,20,30,32], as well as different diameters and lengths of fiber [6,7,8,20,30] were investigated, as was the quantitative aspect [6,7,8,11,13,30,31,32] of the influence of the characteristics of the fiber (glass fiber) and its type on properties of the resulting fiber-reinforced concrete composites. In [10,21,29,31,42], fiber–concrete specimens were studied using high-precision microscopic equipment. However, we note that phenomenologically, we first applied the method of assessing the operation of a fiber from the point of view of studying its deformative characteristics, that is, the perception of fiber as a reinforcing element. That is, a method is proposed for reproducing the macro-level operation of reinforcing elements in building composites at the micro level, that is, drawing an analogy between reinforcing rods in traditional types of concrete and reinforced concrete and fiber, reinforcing primarily a cement matrix and, secondly, a concrete composite.

Thus, the methodology proposed in this article made it possible to assess the quantitative and qualitative aspects of the joint work of fiber and concrete and showed the picture that occurs during the mechanisms of creation, operation, and destruction of such a composite. In quantitative terms, this work was evaluated according to the following indicators: cube strength, prismatic strength, axial tensile strength, flexural tensile strength, ultimate deformations under axial compression, ultimate deformations under axial tension, and elastic modulus. As a result of the assessment, the most rational aspects of joint work were identified, which led to increases in values (Δ, %) (Table 9).

During the manufacture and molding of experimental fiber-reinforced concrete samples, the greatest segregation of the fiber-reinforced concrete mixture was observed in monodispersion-reinforced fiber-reinforced concrete, made using glass fiber with dimensions d = 9 μm and l = 6 mm, and the smallest in polydispersion-reinforced fiber-reinforced concrete. At the same time, the workability of the mixture was maintained constant (P1, draft of the cone 1–4 cm). To reduce or eliminate the segregation of the fiber-reinforced concrete mixture when using this method for field application, the use of plasticizing additives is recommended.

Analysis of the results obtained in the study in the course of comparison with the results obtained earlier by other authors [1,2,3,4,5,6,7,8,9,10,11,12,13,14,15,16,17,18,19,20,21,22,23,24,25,26,27,28,29,30,31,32] revealed a close correlation between the identified quantitative and qualitative aspects and microscopic examination of the structure of the emerging system “fiber–cement matrix–their structure” and, ultimately, the connection of the system “dispersion fiber and its qualitative and quantitative characteristics–heavy fiber-reinforced concrete and its qualitative and quantitative characteristics”. It should be noted that, in this way, the existing ideas of the authors [1,2,3,4,5,6,7,8,9,10,11,12,13,14,15,16,17,18,19,20,21,22,23,24,25,26,27,28,29,30,31,32] who previously studied fiber-reinforced concretes according to various criteria have been developed: the type of fibers, the dispersion of fibers, the aggregated distribution of fibers, the qualitative characteristics of fibers, and the dependence of the properties of fibers and concretes in general.

## 5. Conclusions

A new methodological approach is proposed that considers the “dispersion fiber–cement matrix” system as a microsystem, similar to the “reinforcing rod–concrete matrix” macrosystem. The obtained fundamental new knowledge about the processes occurring during the operation of dispersion fiber in reinforced concrete is presented, which consists of studying the processes of destruction and deformation of fiber, based on computational studies, numerical studies, and microscopic analyses.

Analysis of literature data, as well as data obtained during our own experiments, allows us to formulate the following main conclusions:

- Monodispersion-reinforced fiber-cement specimens made using type B fibers have higher values of compressive strength (up to 12%) and tensile strength in bending (up to 70%) in comparison with fiber-cement specimens reinforced with type A fibers;
- The best values of the adhesion index are observed with the volumetric fiber content in the amount of 2% of the cement mass, regardless of the type of dispersion-reinforcing fiber;
- The microstructure of polydispersion-reinforced fiber-cement specimens is denser, and microcracks formed in the process of destruction of specimens have a smaller opening width (from 60 to 130%) in polydispersion-reinforced specimens;
- Polydispersion-reinforced concretes have higher values of strength (up to 126%) and deformation (up to 296%) characteristics in comparison with monodispersion-reinforced fiber-reinforced concrete.

Thus, the main goal of the study was achieved; the main qualitative and quantitative aspects of the joint work of concrete and fiber with monodispersed and polydispersed reinforcement were determined and their results were evaluated, which are proposed to be used in the technology of new highly functional concrete with improved strength and deformation characteristics, designed to gradually displace traditional reinforced concrete.

Prospects for further research based on the results of the analysis are seen in the study of new theoretical and practical dependencies that arise during the formation of the structure, the establishment of properties, and the change in the nature of the work of various fiber-reinforced concretes under various types of stress–strain states, made from various components and operated in various conditions, for even greater universalization of the proposed concept and methodology.

## Figures and Tables

**Table 1:** Physical and mechanical characteristics of Portland cement PC 500 D0 and its chemical composition.

**Indicator Title**	**Value**
Physico-Mechanical Aspects	
Compressive strength at the age of 28 days, MPa	54.8
Setting time, min	
–start	155
–end	220
Fineness of grinding, passage through a No. 008 sieve, %	96.7
Specific surface area, m^2^/kg	331
Normal density of cement paste, %	23.5
Chemical	
Weight loss on ignition, %	0.70
Silicon oxide content (SiO_2_), %	20.89
Aluminum oxide content (Al_2_O_3_), %	4.72
Iron oxide content, (Fe_2_O_3_), %	4.32
Calcium oxide content (CaO), %	63.27
Magnesium oxide (MgO), wt %	2.45
Sulfuric acid anhydride (SO_3_), wt %	2.81
Alkaline oxides in terms of Na_2_O, wt %	0.69
Free calcium oxide content (CaO_fr_), %	0.00
Chloride ion (Cl^−^), wt %	0.038
Insoluble residue, %	0.20

**Table 2:** Physical and mechanical characteristics of aggregates.

Aggregates	Grain Composition (Fraction, mm/ Sieve Size, mm Partial and Full Sieve Rest, %, Fineness Modulus)						Bulk Density, kg/m^3^	True Density, kg/m^3^	Crushing, wt %/ Content of Dust and Clay particles, %	Content of Lamellar and Needle-Shaped Grains, wt %	Voids, %
	2.5	1.25	0.63	0.315	0.16	<0.16					
Crushed stone	5–20						1437	2620	11.4	8.1	45
Sand	0.17	1.39	8.86	45.8	41.0	2.49	1438	2650	1.1	-	-
	0.17	1.56	10.4	56.2	97.3	99.7					
	1.66										

Note: sign “/” separates the characteristics for crushed stone and sand, respectively

**Table 3:** Physical and mechanical properties of fiber.

Glass fiber	Tensile Strength, MPa	Fiber Diameter, μm	Fiber Length, mm	Elastic Modulus, GPa	Density, kg/m³	Elongation to Break, %
A	3100	9	6	72	2.6	4.6
B		13	12			

**Table 4:** Amount of fiber content adopted by earlier researchers.

Research Sources	Fiber Material	Volumetric Content of Fibers wt, % (Min–Max)
[1,2]	amorphous metallic	0.1–1.0
[3]	basalt	0.45–1.6
[4]	steel	0.5–2.5
[6]	steel	0.25
[7]	polypropylene basalt, glass, mineral wool, steel, amorphous	0.1–1.0
		1.0–3.0
[8]	glass, carbon, aramid	0.5–2.0
[10]	steel, basalt polypropylene	1.5 1.25
[14]	steel ultrashort ultrafine steel polypropylene	1.92 4.0 0.27
[17]	steel	2
[18]	Kevlar + carbon	1.0
[19]	nylon + jute	1.0
[20]	steel, polypropylene	0.25–0.45
[21]	PVA steel	1.0–2.0 1.5–4.0
[30]	glass, basalt	0.25–2.0
[31]	glass	0.5–3.0
[32]	glass, polypropylene	0.5–2.0

**Table 5:** Experimental research program.

Series Number	Volumetric Content of Fibers with d = 9 μm, l = 6 mm, wt %	Volumetric Content of Fibers with d = 13 μm, l = 12 mm, wt %
C	-	-
1A	1	-
2B	-	1
3AB	0.5	0.5
4A	2	-
5B	-	2
6AB	1	1
7A	3	-
8B	-	3
9AB	1.5	1.5
10A	4	-
11B	-	4
12AB	2	2

**Table 6:** Characteristics of adhesion of fibers to cement stone for monodispersion-reinforced specimens.

**Series Number**	**Adhesion Indicator (τ, MPa)**
1A	0.17
2B	0.13
4A	0.18
5B	0.17
7A	0.17
8B	0.15
10A	0.12
11B	0.10

**Table 7:** Parameters of the composition of the concrete mixture.

Indicator Title	W/C	C, kg/m^3^	W, l/m^3^	CS, kg/m^3^	S, kg/m^3^	ρ_cm_, kg/m^3^
Indicator value	0.58	327	190	1315	573	2405

W/C is water/cement ratio; C is Portland cement; W is water; CS is crushed stone; S is sand; ρ_cm_ is concrete mixture density.

**Table 8:** Test results of concrete and fiber-reinforced concrete samples for strength and deformation characteristics.

Concrete Characteristics	Heavy Concrete	Monodispersion-FRC (d = 9 μm, l = 6 mm)	Monodispersion-FRC (d = 13 μm, l = 12 mm)	Polydispersion-FRC
R_b,cub_, MPa	59.8	61.8	62.7	65.5
R_b_, MPa	44.8	46.3	47.1	49.9
R_btb_, MPa	7.3	12.5	14.1	16.5
R_bt_, MPa	4.2	5.2	5.7	6.1
ε_bR_, mm/m × 10^−3^	2.09	2.67	2.75	2.86
ε_btR_, mm/m × 10^−4^	1.23	3.11	4.54	4.87
E, GPa	38.9	40.2	41.4	43.5

**Table 9:** Increases in strength and deformation characteristics of fiber-reinforced concrete specimens depending on the type of dispersed reinforcement.

Concrete Characteristics	Δ, % in Relation to Heavy Concrete (Monodispersion-Fiber-Reinforced Concrete)		
	Monodispersion-FRC (d = 9 μm, l = 6 mm)	Monodispersion-FRC (d = 13 μm, l = 12 mm)	Polydispersion-FRC
R_b,cub_, MPa	+3.3	+4.8	+9.5 (+4.5)
R_b_, MPa	+3.2	+5.1	+11 (+5.9)
R_btb_, MPa	+71	+93	+126 (+17)
R_bt_, MPa	+24	+36	+45 (+7.0)
ε_bR_, mm/m × 10^−3^	+28	+32	+37 (+4.0)
ε_btR_, mm/m × 10^−4^	+153	+269	+296 (+7.3)
E, GPa	+3.3	+6.4	+12 (+5.1)

## Data Availability

The study did not report any data.

## Supplementary Materials

The following supporting information can be downloaded at: https://www.mdpi.com/article/10.3390/polym14040682/s1, Figure S1: The nature of the destruction of samples: (a) unreinforced concrete; (b) fiber-reinforced concrete.
