# Supplementary material for: Quantitative and Qualitative Aspects of Composite Action of Concrete and Dispersion-Reinforcing Fiber
Source: Polymers (Basel). 2022 Feb 11;14(4):682. doi: 10.3390/polym14040682 (PMC8876351; doi:10.3390/polym14040682)
Supplement: Supplementary file 1 [file polymers-14-00682-s001.zip › polymers-1567425-supplementary.pdf]

Supplementary Materials:

Quantitative and Qualitative Aspects of Composite Action of Concrete and Dispersion-Reinforcing Fiber

**Sergey A. Stel'makh [1], Evgenii M. Shcherban' [1], Alexey Beskopylny [2,*], Levon R. Mailyan [3], Besarion Meskhi [4]
and Valery Varavka [5]**

[1] Department of Engineering Geology, Bases, and Foundations, Don State Technical University,
344003 Rostov-on-Don, Russia; sergej.stelmax@mail.ru (S.A.S.); au-geen@mail.ru (E.M.S.)
[2] Department of Transport Systems, Faculty of Roads and Transport Systems, Don State Technical University, 344003 Rostov-on-Don, Russia
[3] Department of Roads, Don State Technical University, 344003 Rostov-on-Don, Russia; lrm@aaanet.ru
[4] Department of Life Safety and Environmental Protection, Faculty of Life Safety and Environmental Engineering, Don State Technical University, 344003 Rostov-on-Don, Russia; reception@donstu.ru
[5] Research and Education Center "Materials", Don State Technical University, Gagarin sq., 1, 344003 Rostov-on-Don, Russia; varavkavn@gmail.com
* Correspondence: besk-an@yandex.ru; Tel.: +7-8632738454

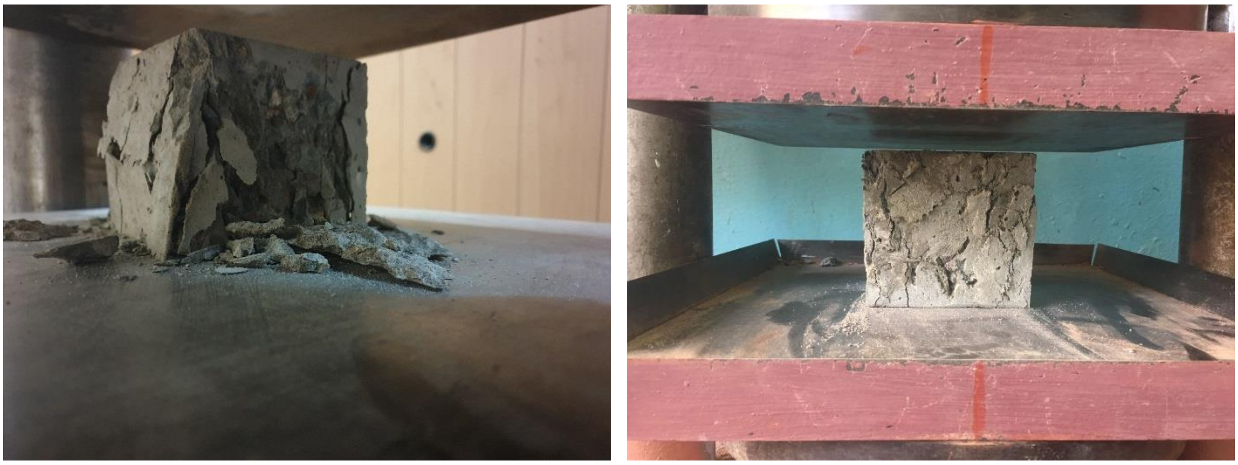

(**a**) (**b**)

Figure S1. The nature of the destruction of samples: (**a**) unreinforced concrete; (**b**) fiber-reinforced concrete
